# Glucose restriction decreases telomerase activity and enhances its inhibitor response on breast cancer cells: possible extra-telomerase role of BIBR 1532

**DOI:** 10.1186/1475-2867-14-60

**Published:** 2014-07-04

**Authors:** Layal Wardi, Nada Alaaeddine, Issam Raad, Riad Sarkis, Rim Serhal, Charbel Khalil, George Hilal

**Affiliations:** 1 Cancer and Metabolism Laboratory, Faculty of Medicine, Campus of Medical Sciences, Saint-Joseph University, Damascus Road, P.O.Box 11-5076, Riad el Solh, Beirut 1107 2180, Lebanon; 2 Regenerative Medicine Laboratory, Faculty of Medicine, Saint-Joseph University, Beirut, Lebanon; 3 Department of Infectious Diseases, the University of Texas M. D. Anderson Cancer Center, Houston, TX, USA; 4 Surgery Department, Faculty of Medicine, Saint-Joseph University and Hotel-Dieu de France, Beirut, Lebanon

**Keywords:** Glucose restriction, Cancer, Telomerase, Breast cancer, BIBR 1532

## Abstract

**Background:**

Considerable progress has been made to understand the association between lifestyle and diet in cancer initiation and promotion. Because excessive glucose consumption is a key metabolic hallmark of cancer cells, glucose restriction (GR) decreases the proliferation, and promotes the differentiation and transformation of cancer cells to quiescent cells. The immortality of cancerous cells is largely assured by telomerase, which is an interesting target for inhibition by BIBR 1532. In this study, we investigated the effect of GR on telomerase activity and on the efficacy of its inhibition by BIBR 1532.

**Methods:**

Breast cancer MDA-MB 231 and MCF-7 cells were cultured in DMEM (Dulbecco’s modified eagle’s media) with 0, 1 or 4.5 g/l of glucose. The telomerase activity was measured via quantitative Real-Time PCR, and the two telomerase subunits were semi-quantified by RT-PCR. Proliferation test and mitochondrial metabolism were assessed via tetrazolium salt reduction and cell counts; apoptosis was assessed via caspase-3 quantification and flow cytometry.

**Results:**

A decrease in the telomerase activity of more than 75% was associated with a significant reduction in the mRNA expression of its catalytic subunit hTERT (Reverse Transcriptase) and a decrease in the mitochondrial metabolism by more than 80% under restricted glucose conditions. In addition, GR increased the effect of BIBR 1532. Glucose deprivation induces apoptosis via BIBR 1532-mediated telomerase inhibition in triple negative breast cancer cells, as assessed by caspase-3 measurements and Annexin analysis.

**Conclusions:**

Taken together, our results suggest that the effect of BIBR 1532 is potentiated by GR to induce triple negative breast cancer cell death.

## Background

Caloric restriction (CR) or reducing the total caloric intake by 20 to 40% without limiting essential vitamins and minerals has long been considered as a means to prevent and cure cancer and to extend life span [1-3]. Recently, a potential role of CR in the treatment and intervention of age-related diseases, including cardiovascular disease, diabetes, hypertension, atherosclerosis and cancer, has been investigated [4,5].

Cancer is a complex disease associated with multiple DNA mutations that lead to abnormal cell proliferation, immortality and tissue invasion at distant sites [6]. The strong relationship between nutrition and cancer, as also demonstrated by several epidemiological studies [7,8], has been extrapolated by some clinicians into complete abstinence from food. In fact, ten oncologists did allow their patients who suffered from breast, esophageal, prostate and lung cancer to fast from 48 to 140 hours pre-chemotherapy and 5 to 56 hours post chemotherapy [9]. Patients who received chemotherapy with water-only fasting reported a reduction in side effects and also showed a better response to treatment than the control group. This latter observation contradicts the dietary recommendation of the American Cancer Society that advises patients to increase their protein and calorie intake before chemotherapy administration.

Cell division is potentially a risky process, and cell division is limited in normal tissues. Telomerase is up-regulated in 85% of malignant tumors. Therefore, they escape senescence to have unlimited cell division at the earlier stage of tumorogenesis [10]. Telomerase is the key enzyme responsible for the immortalization of cancerous cells [11].

Telomerase is a ribonucleoprotein complex enzyme that consists of two major subunits, the human Telomerase Reverse Transcriptase (hTERT) and human RNA component (hTR), in addition to several accessory proteins that modulate the activity of this enzyme [12]. Eighty five percent of malignant tumors escape senescence to have unlimited cell division following telomerase up-regulation during the early stages of tumorogenesis [10]. The enzyme telomerase stabilizes the length of telomeres, the end of linear chromosomes, via the *de novo* addition of telomeric repeats, which have been lost during incomplete DNA replication, telomere degradation or oxidative stress. The telomerase-induced telomere maintenance and reparation is believed to play a critical role in senescence, aging and cancer [13]. Human telomerase consists of two major subunits, the human Telomerase Reverse Transcriptase (hTERT) and human RNA component (hTR), in addition to several accessory proteins that modulate the activity of this enzyme [12].

An increasing body of literature has demonstrated that several metabolic changes occur during cancer evolution [14]. On the molecular level, cancer cell proliferation is tightly related to cell metabolism [15]. In multicellular organisms, cell proliferation is controlled, and any aberrant individual cell proliferation is prevented when nutrients exceed the levels needed to support cell division. In contrast, cancer cells overcome this growth factor control via several DNA mutations that alter the receptor-initiated signaling pathway [16,17]. This altered metabolism of tumor cells is exhibited by an increased glucose uptake and elevated aerobic glycolysis, which was first recognized by Warburg 70 years ago [18]. Unsurprisingly, abnormal glucose metabolism has become the target of several studies because of its uniqueness in cancer cells and its possible role in preventing and curing cancer. However, GR is a metabolic stressor to many signaling pathways, including the anti-apoptotic pathway IGF1R-IR/PI3K/Akt/mTOR [19]. Glycolytic enzymes are up-regulated following the activation of this pathway. In addition to glycolysis activation, c-Myc and SP1, which are transcription factors induced by a high glucose level, cooperate to activate the transcription of the catalytic subunit of telomerase (hTERT) [20] The activation of AMP-activated protein kinase by GR down-regulates mTOR, glycolysis activity, and cell proliferation [21-23]. Furthermore, glucose withdrawal increases ketone bodies in the blood circulation. These ketone bodies can reportedly help to prevent and treat cancer by protecting mitochondria from inflammation and ROS (Reactive Oxygen Species) [24]. Finally, Li and colleagues [25] demonstrated that GR impairs precancerous cell growth via the epigenetic control of the telomerase subunit (hTERT) and p16 expression.

GR is an attractive modality to explore because it has been shown to induce changes in molecular pathways, especially those altered in cancer. Altering these pathways could make cells more susceptible to treatment with the telomerase inhibitor BIBR 1532. This drug is a potent and selective inhibitor of two telomerase units; it interferes with the action of telomerase [26].

The primary objective of this article was to investigate the effect of GR on the telomerase activity and on its inhibition by BIBR1532 in two breast cancer cell lines, the highly invasive and metastatic triple-negative (ER-, PR-, Her2-) MDA-MB 231 and the less invasive Estrogen-dependent (ER+) MCF-7 cells.

## Results

### Effect of glucose restriction (GR) on telomerase activity

**Figure 1:**
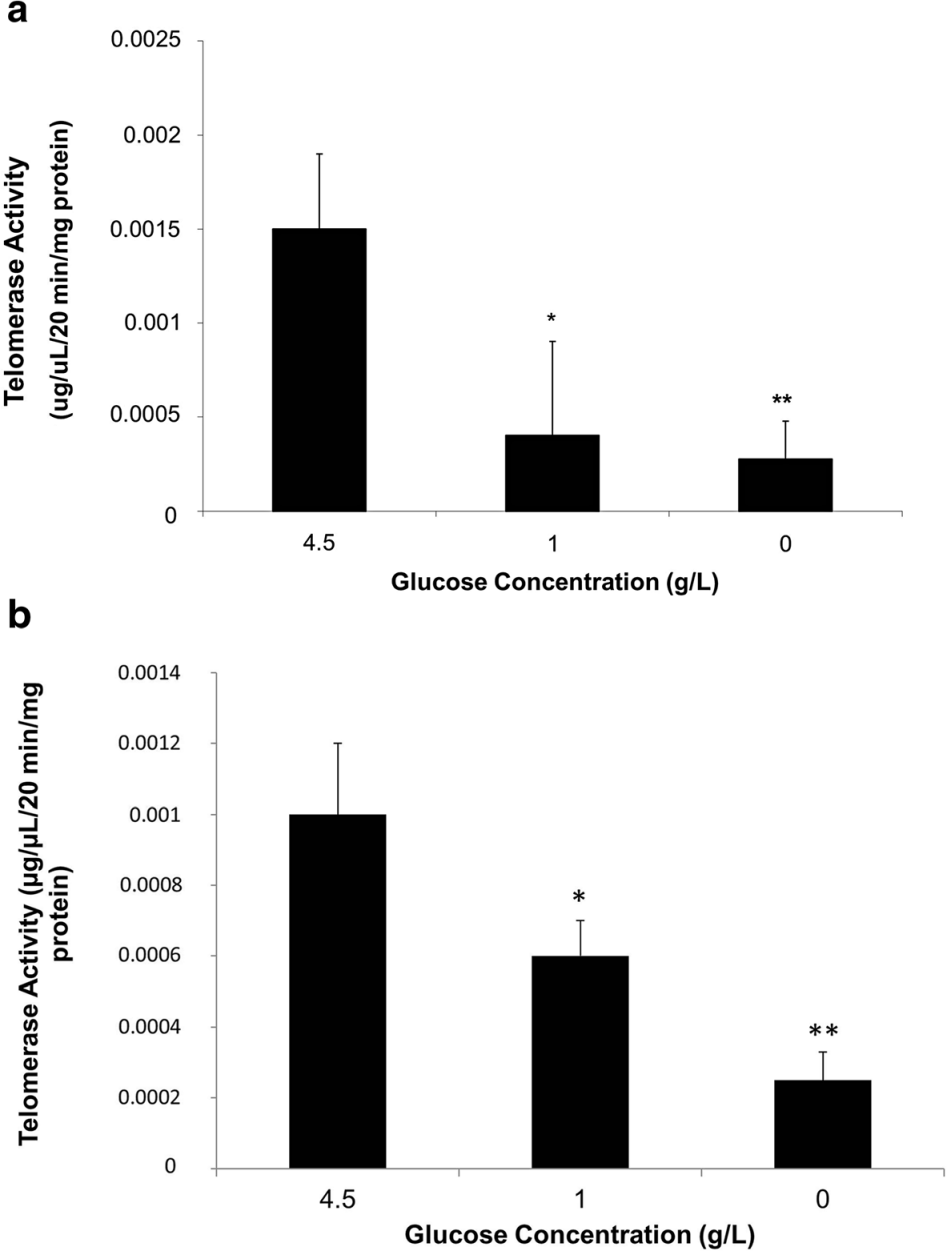
**Effect of GR on the telomerase activity. a**. MDA-MB 231 and **b**. MCF-7 cells were seeded in 75 cm^2^ flasks and grown in DMEM culture medium containing 10% FBS and 1% Pen/Strep at different glucose concentrations (0, 1 and 4.5 g/L of glucose). After 5 days of pre-incubation in the appropriate medium 1.5 million cells of each condition were prepared for the telomerase activity and protein measurements. The bars represent values from at least three independent experiments (*P < 0.05, **P < 0.01).

The effect of GR on the telomerase activity was first assessed using the MDA-MB 231 and MCF-7 breast cancer cell lines. Figure 1 shows that decreasing the glucose concentration in culture media led to a partial telomerase activity inhibition at 1 and 0 g/l. Importantly, the cells incubated with 0 g/l of glucose were cultured in 10% of FBS that contains 0.65 g/l of glucose. This concentration was measured for each test in a medical laboratory and mimics physiological glycaemia in fasting situations. MDA-MB 231 reduced their telomerase activity in response to GR by more than 50% (P < 0.05) and 80% (P < 0.01) at 1 g/l and 0 g/l of glucose, respectively. This same pattern was evident in MCF-7 cells, as indicated in Figure 1b. The telomerase activity decreased by 40% and 75% in cells incubated in DMEM containing 1 g/l (P < 0.05) and 0 g/l glucose (P < 0.01), respectively.

### Glucose restriction but not BIBR 1532 impaired hTERT expression

**Figure 2:**
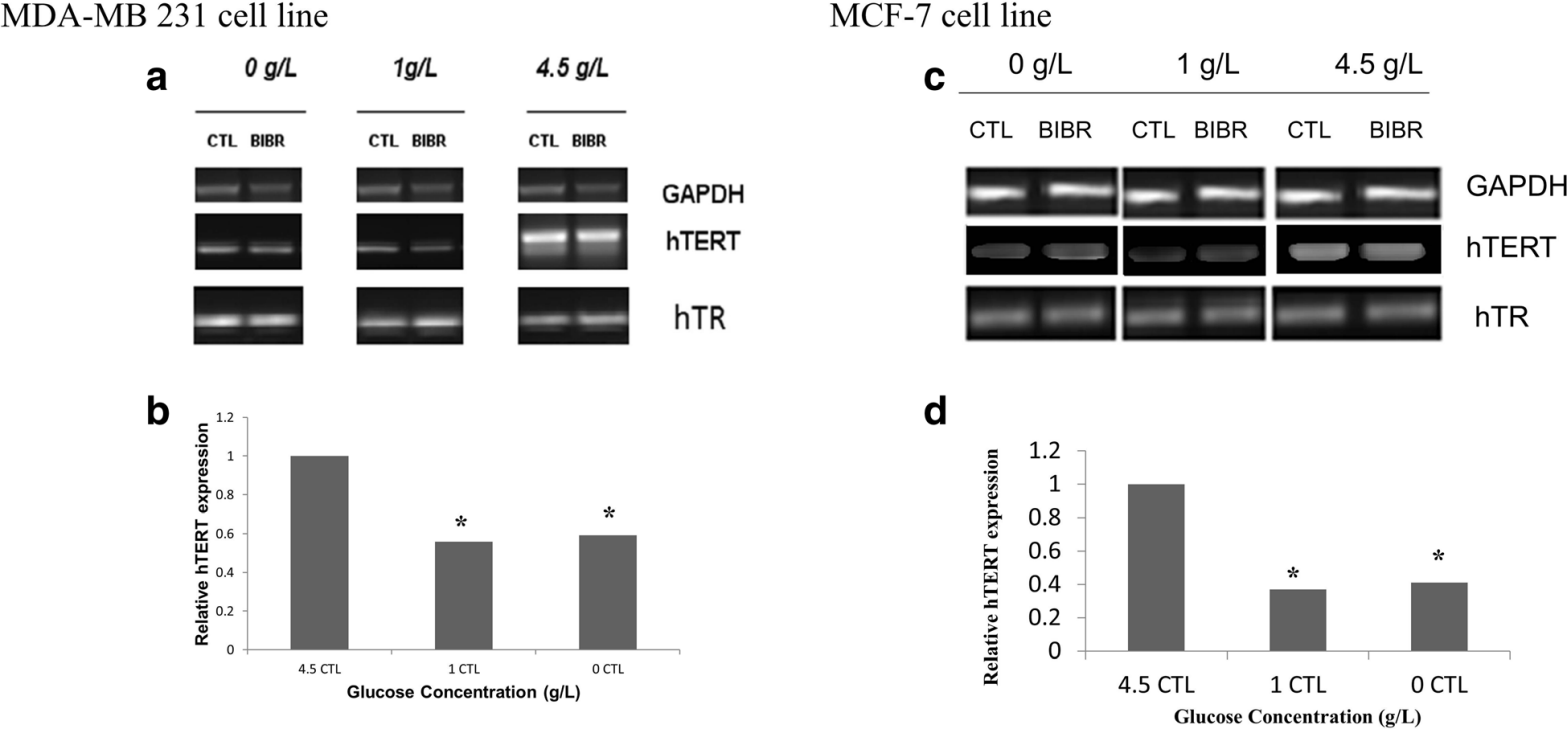
**Regulation of hTERT mRNA expression by 10 μM BIBR 1532 in MDA-MB-231 and MCF-7 cells.** Cells grown in different glucose concentrations were treated with telomerase inhibitor for 48 h. The effect of 10 μM BIBR 1532 on gene expression was examined by RT-PCR. Aliquots of each reaction were equalized as shown by the internal control of GAPDH. **a** and **c** show the effect of GR and BIBR 1532 treatment on the hTERT mRNA expression in MDA-MB 231 and MCF-7, respectively. The relative expression levels of hTERT at different glucose concentrations are shown in **b** and **d**. The result presented represents three independent experiments. (*P < 0.05, **P < 0.01).

Because the hTR component is constitutively expressed in all cells and hTERT is up-regulated in most tumor cells, we investigated the ability of GR to modulate the expression of hTERT in human breast cancer cell lines after 48 h of treatment with 10 μM BIBR1532. The RT-PCR method was used to examine the expression of both hTR and hTERT mRNA using GAPDH as an internal control. Importantly, BIBR 1532 inhibits the protein, but not the mRNA level of hTERT; it inhibits the mechanism of action of the enzyme.The relatively high telomerase activity at high glucose concentrations shown in Figure 1 was confirmed by high mRNA expression (Figure 2). However, we could not demonstrate any changes in the expression of hTERT and hTR mRNA in cells grown in 1 and 0 g/l of glucose; hTERT was expressed at 55% (P = 0.0248) and 59% (P = 0.033) at 1 and 0 g/l of glucose, respectively, in MDA-MB 231, while hTERT was expressed at 41% (P = 0.021) and 37% (P = 0.0178) in 0 g/L and 1 g/L, respectively, in MCF-7 cells, as shown in Figure 2c. As expected, BIBR 1532 did not affect the telomerase transcription because it acts directly on the protein.

### Glucose restriction decreased cell proliferation and enhanced the effect mediated by BIBR 1532

**Figure 3:**
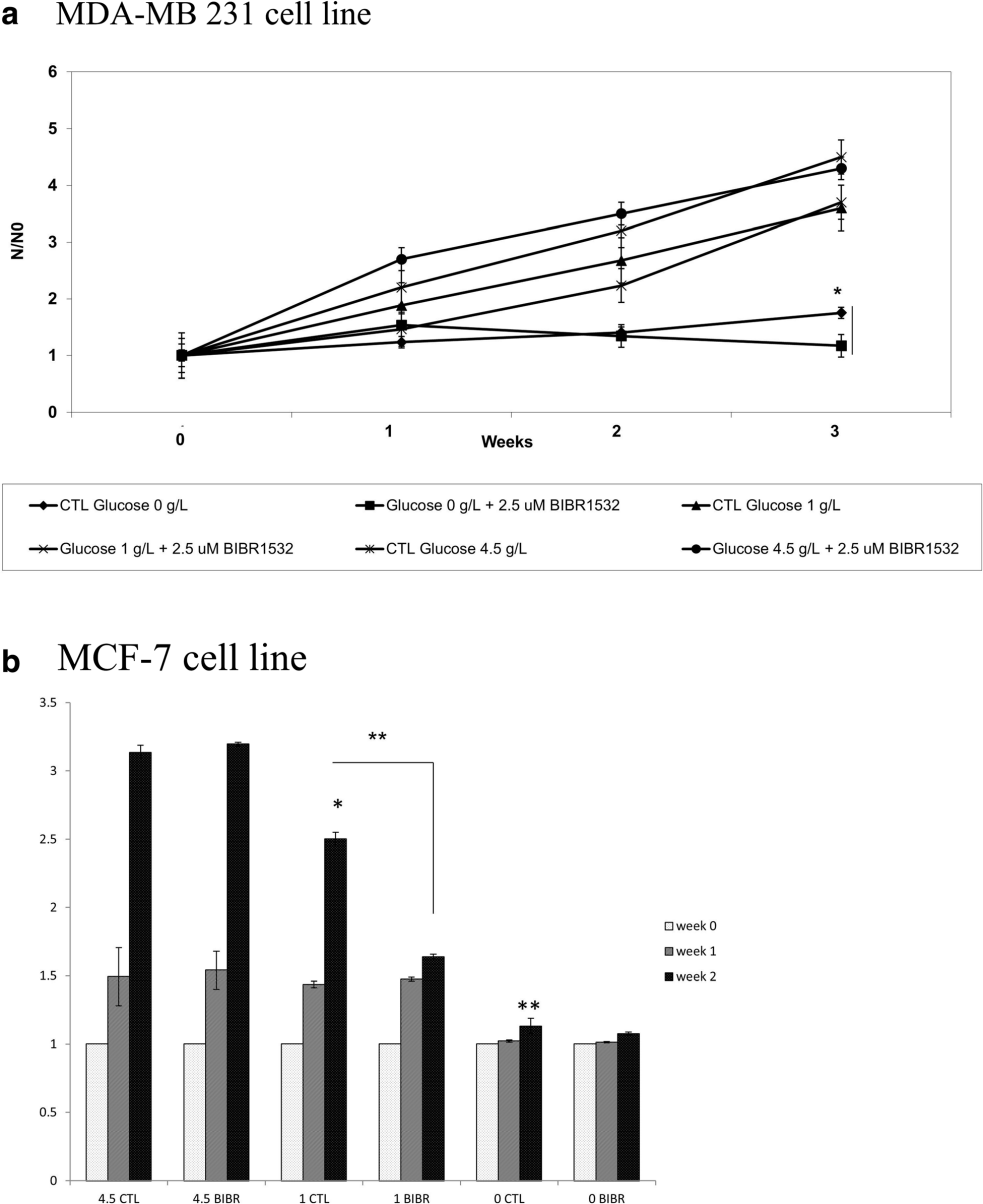
**Effect of GR on the proliferation of MDA-MB 231 and MCF-7 BIBR 1532 treated cells.** The cells were seeded at a very low density in a 25 cm^2^ flask and grown in DMEM culture medium containing 10% FBS and 1% Pen/Strep at different glucose concentrations with or without 2.5 μM of BIBR 1532. The medium was replaced every two days, and fresh telomerase inhibitor was added each time. At the end of each week, the cells were split and counted at least three times using bromophenol blue and a hemocytometer. **a** and **b** show the effect of GR and treatment with 2.5 μM BIBR 1532 on MDA-MB 231 and MCF-7 cells based on the obtained cell number after each count compared to the number seeded cells. Bars represent values from at least three independent experiments (*p < 0.05, *p < 0.01).

The effect of GR on the MDA-MB 231 cell count and proliferation was only significant at 0 g/l of glucose compared to 4.5 g/l of glucose, as shown in Figure 3a. Although cell proliferation at 1 g/l of glucose seems to be lower compared to cells grown in 4.5 g/l of glucose, the results did not reach significance (P = 0.08). In contrast, the proliferation of cells in 0 g/l of glucose was very slow, and this difference was statistically very significant compared to those incubated in 1 and 4.5 g/l of glucose (P < 0.001). The effect of the telomerase inhibitor BIBR 1532 was very pronounced for cells grown in 0 g/l of glucose. By the end of week three, the number of cells incubated in 0 g/l of glucose and 2.5 μM BIBR 1532 was almost zero (Figure 3a). Contrary to this result, BIBR 1532 seemed to have an effect in weeks one and two for cells grown in 1 and 4.5 g/l of glucose. This trend disappeared at week three, and the level of cell proliferation was similar to that of the control.The effect of GR was more pronounced on the MCF-7 cell line. As shown in Figure 3b, reducing the glucose concentration in the media significantly decreased the cell proliferation over time. The proliferation of cells grown without glucose reduced by 30% from the first week (P = 0.057), and this reduction was enhanced at the second week, when only less than 40% of cells remained alive (P = 0.0001). The MCF-7 cell count was not extended into the third week because all of the cells grown in DMEM containing 0 g/L of glucose had died. Cells incubated in 1 g/L of glucose exhibited reduced proliferation by 20% at the second week (P = 0.0139). After 15 days, 2.5 μM BIBR 1532 could reduce cell proliferation by 35% only when cells were incubated in physiological glucose concentrations (P = 0.0051).

### Regulation of mitochondrial metabolism by glucose restriction and BIBR 1532

**Figure 4:**
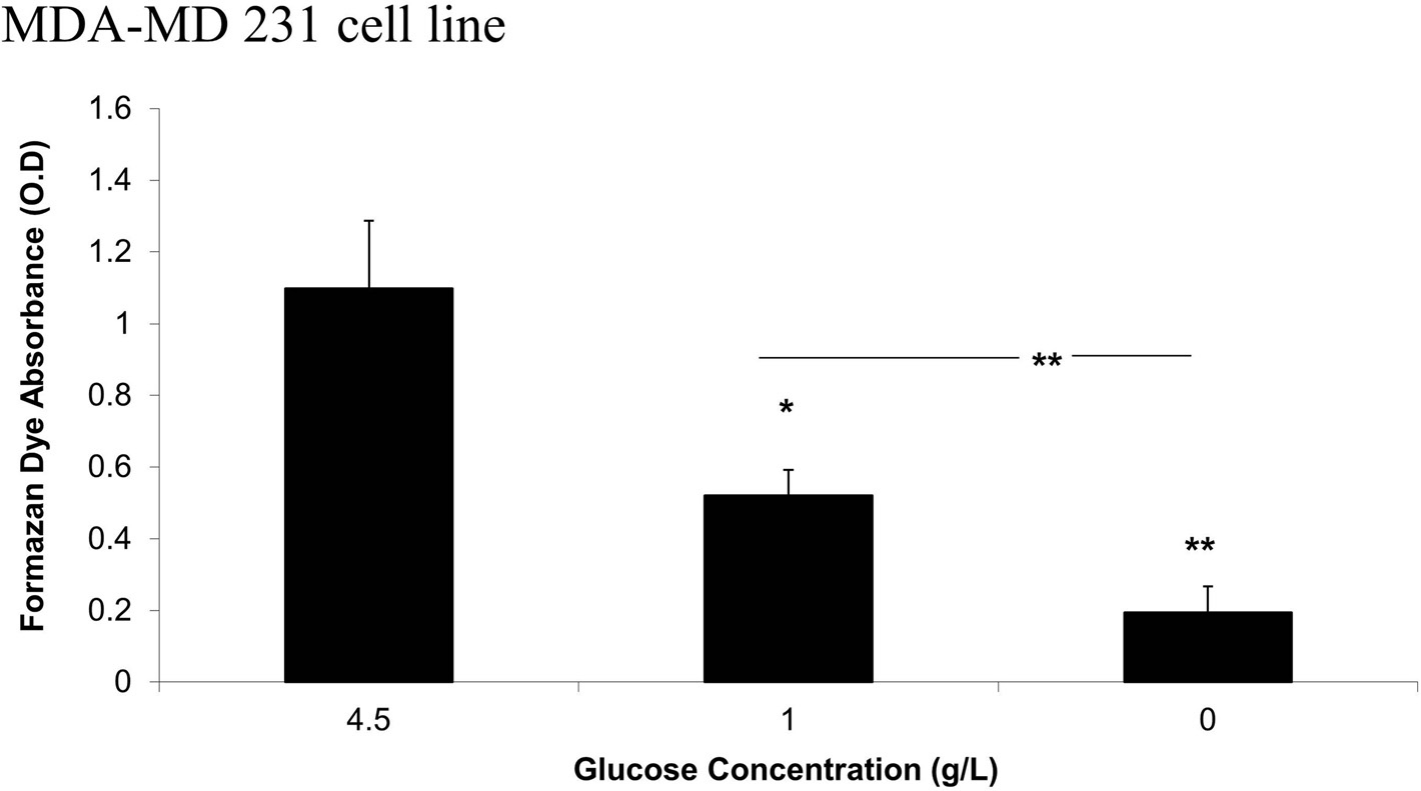
**Effect of GR on cell viability and mitochondrial metabolism.** The MDA-MB 231 cells grown in DMEM with different glucose concentration were equally seeded in 96-well plates at a density of 10^4^ cells/well. Twenty-four hours later, cells received 10 μl of tetrazolium salt and were incubated for 60 minutes at 37°C. The formation of formazan dye was assessed using an ELISA reader at 450 nm. The O.D. of each condition is a mean of 12 wells. Bars represent values from at least three independent experiments. (*p < 0.05 and **p < 0.01).

**Figure 5:**
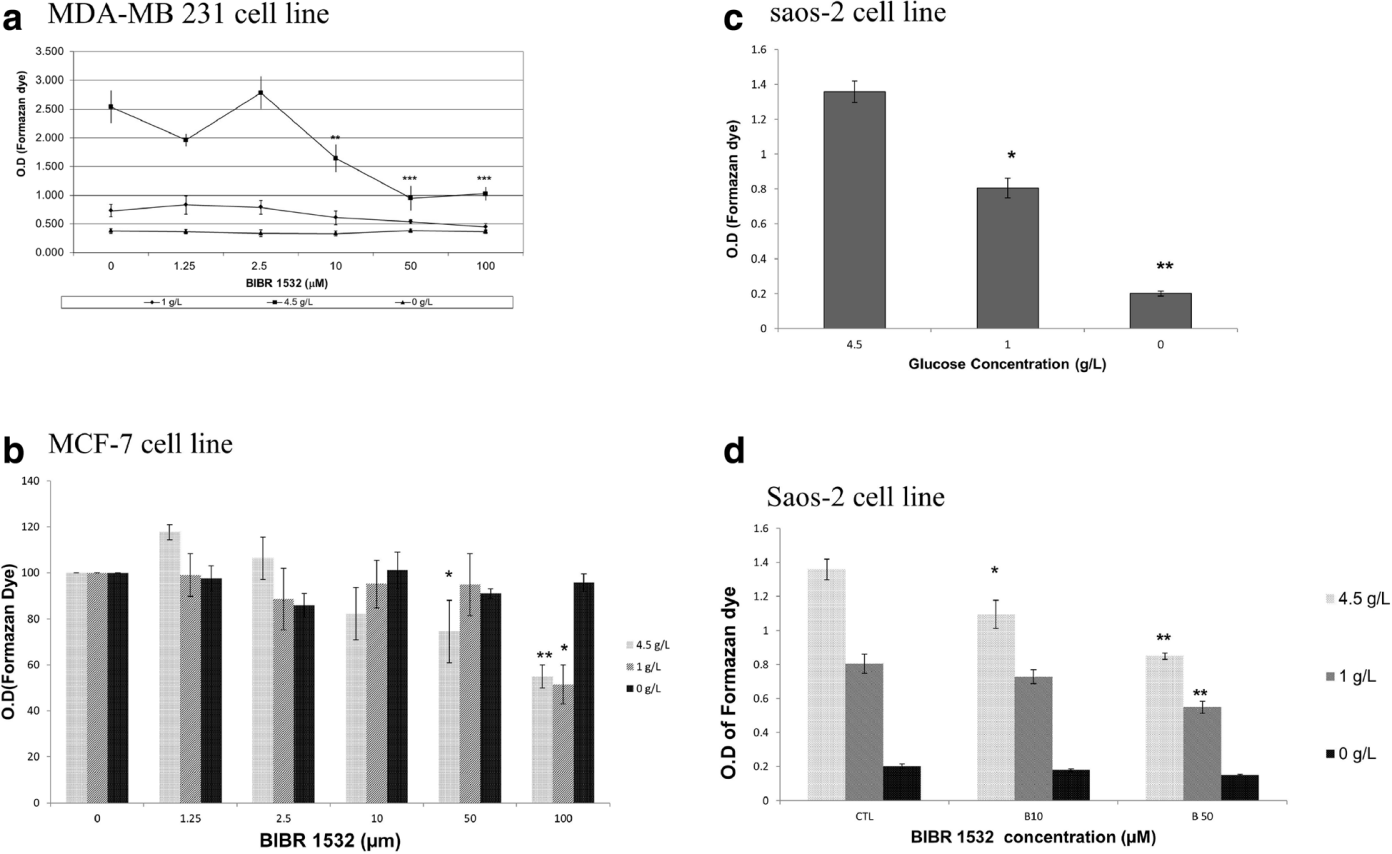
**Proliferation test of MDA-MB 231, MCF-7 and of saos-2 cells.** Cells grown in DMEM with different glucose concentrations were equally seeded in 96-well plates at a density of 10^4^ cells/well. Twenty-four hours later, the cells were treated with different concentrations of BIBR 1532 for 48 hours. At the end of treatment, each well received 10 μl of tetrazolium salt and was incubated for 60 minutes at 37°C. The formation of formazan dye was assessed using an ELISA reader at 450 nm. **a** and **b** represent the dose response curves of MDA-MB 231 and MCF-7 to BIBR 1532, respectively. **c** represents the effect of GR, while **d** represents the effect of BIBR 1532 on the mitochondrial metabolism of saos-2 cell line. The O.D. of each condition is a mean of 12 wells. Bars represent values from at least three independent experiments (*P < 0.05, **P < 0.01).

Water-soluble tetrazolium salt WST-1 was used to assess the cell viability. This test reflects not only the cell viability but also the metabolism of mitochondria, as reported by several works [27,28]. Figure 4a shows that GR apparently decreased the succinate reductase activity in MDA-MB 231 cells by 55% and by more than 5-fold for cells grown in 1 g/l and 0 g/l of glucose, respectively. In MCF-7 cells, GR reduced the mitochondrial activity by 35% and by more than 80% in cells grown in 1 g/l and 0/gl glucose, respectively (P = 0.0009). This decrease was not caused by cell death because healthy quasi-confluent cells were detected in all wells using microscopic examination, bromophenol blue staining, and cell counts. This experiment was extended to evaluate the effect of BIBR 1532 in the GR conditions. As shown in Figure 5a, BIBR 1532 significantly reduced succinate reductase activity in MDA-MB 231 cells grown in 1 g/L and 4.5 g/L glucose. This effect was potentiated at a high glucose level (4.5 g/L), where 10 μM BIBR 1532 could reduce WST-1 transformation by 35%. However, this effect was absent when cells were grown in 0 g/l glucose.As shown in Figure 5b, higher concentrations of BIBR 1532 are required to elicit the same effect observed in MDA-MB 231 cells. This telomerase inhibitor could reduce WST-1 transformation by 25% starting from 50 μM, and this reduction became more significant when the BIBR 1532 concentration was increased to 100 μM (P = 0.0011). A high concentration of BIBR 1532 (100 μM) is required to inhibit the mitochondrial metabolism by ~25% in cells incubated in 1 g/L of glucose (P = 0.029). This modulator effect was absent in cells grown without glucose.Telomerase-negative human osteosarcoma Saos-2 cells have previously been studied to examine the role of telomerase in mitochondrial metabolism modulation. The Saos2 cell line was cultured in different glucose concentrations and then treated with 10 and 50 μM BIBR 1532 over 48 h. The results demonstrated that GR displayed the same profile as those exhibited by the two breast cancer cell lines. Reducing the glucose concentration in the media decreases the WST-1 transformation by 40% and more than 80% for cells grown in 1 g/L and 0 g/L glucose, respectively (Figure 5c). As shown in Figure 5d, BIBR 1532 exhibits the same effect in Saos-2 cells grown in 1 and 4.5 g/L glucose (P = 0.017 and P = 0.0032, respectively).

### hTERT mRNA silencing did not affect mitochondrial metabolism

**Figure 6:**
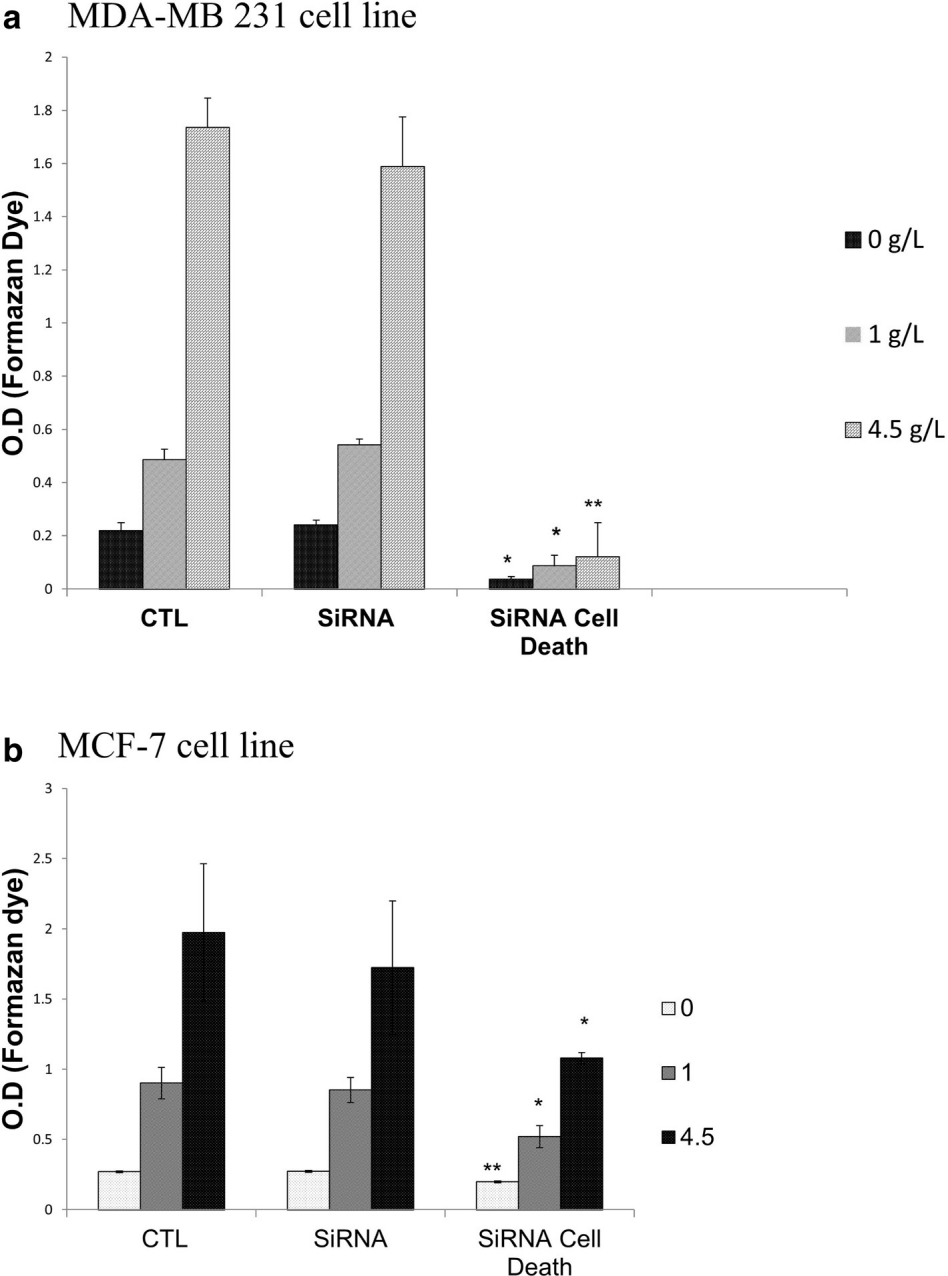
**Effect of telomerase siRNA on cell viability and mitochondrial metabolism.** MDA-MB 231 **(a)** and MCF-7 **(b)** cells grown in DMEM with different glucose concentrations were equally seeded at a density of 2 × 104 in a 96-well plate 10 min after 20 nM SiRNA transfection. Seventy-two hours later, each well received 10 μl of WST-1 and was incubated for 60 minutes at 37°C. The formation of formazan dye was assessed using an ELISA reader at 450 nm. The O.D. of each condition is a mean of 12 well. Bars represent values from at least three independent experiments. (*P < 0.05, **P < 0.01).

To further validate the results concerning the modulation of mitochondrial metabolism by BIBR 1532, we treated MDA-MB 231 and MCF-7 cells grown at different glucose concentrations with TERT siRNA. Surprisingly, Figure 6(a, b) shows that 20 nM TERT siRNA did not affect the formazan dye formation compared to the control at the three different glucose concentrations; however, a decreased tendency was evident only in cells incubated in 4.5 g/l glucose. The transfection efficiency was confirmed by using electrophoresis and cell death control, which is a blend of highly potent siRNAs targeting ubiquitously expressed human genes indispensable for cell survival (Figure 6) and hTERT gene expression. This apparently discrepant result could be explained by a high residual telomerase activity even after partial hTERT mRAN silencing. Furthermore, hTERT siRNA likely did not elicit an effect similar to that of BIBR 1532 due to the insufficient incubation time to deplete hTERT.

### Glucose deprivation induced apoptosis mediated by BIBR 1532

To demonstrate whether BIBR 1532 induced apoptosis in MDA-MB 231 and MCF-7 cells, the expression of caspase-3 was assessed via ELISA (*Invitrogen*).

Although apoptotic signals are believed to be mediated by a hierarchy of caspase activation, only two pathways of caspase activation have been described. Nevertheless, caspase-3 is a common downstream enzyme activated in apoptosis that is induced by most stimuli.

**Figure 7:**
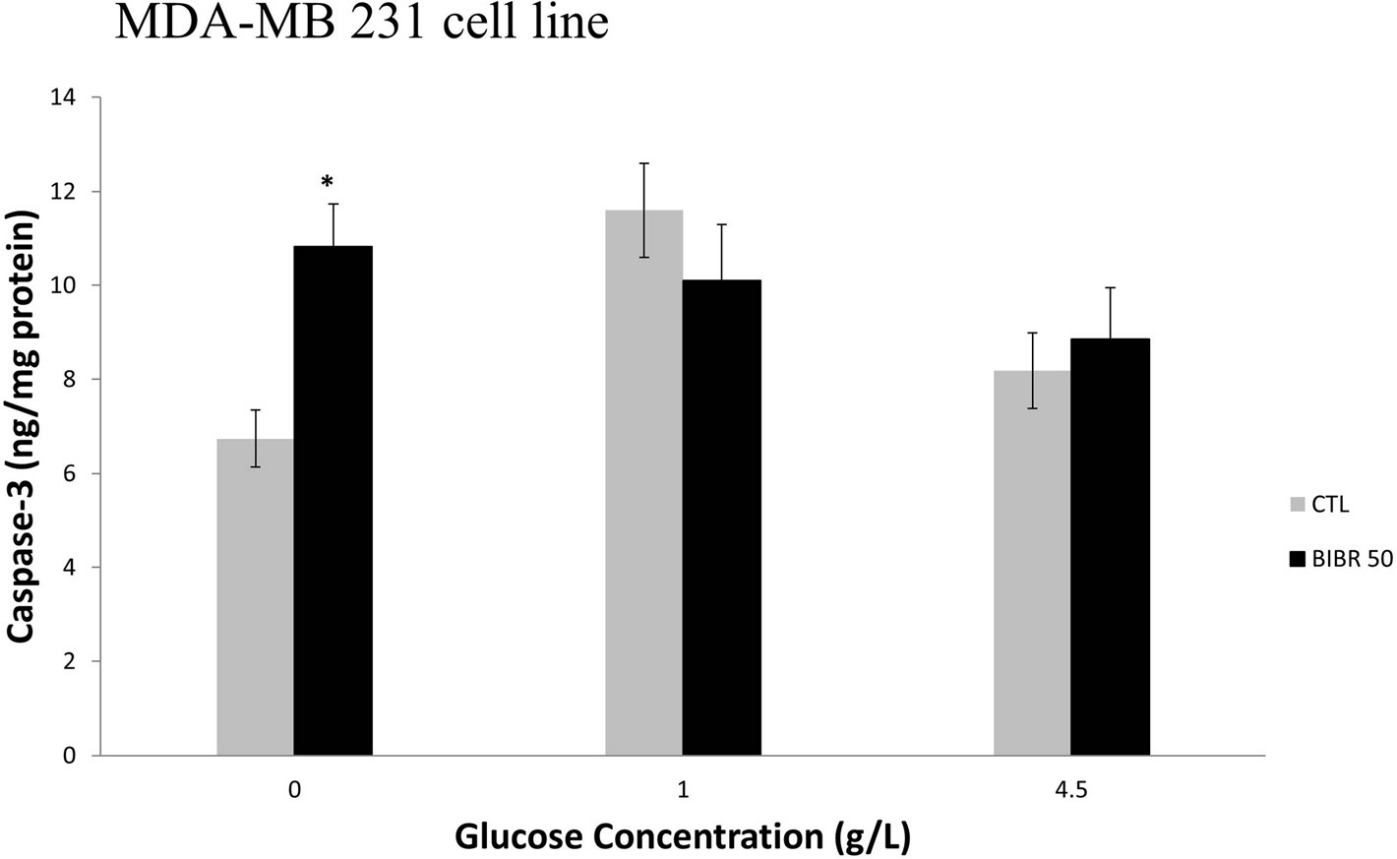
**Effect of high BIBR 1532 concentration on cell apoptosis.** MDA-MB 231 cells grown at different glucose concentrations were seeded in 6-well plates at a density of 2 × 10^5^ cells per well. After adherence, the cells were treated with 50 μM BIBR 1532 for 48 hours. After treatment, the culture media was discarded, and the cells were harvested and lysed as mentioned in materials and methods to further quantify the caspase-3 level via ELISA and measure the protein concentration. Bars represent values from at least three independent experiments (*p < 0.05).

The level of caspase-3 was measured in cells grown at different concentrations of glucose in the presence and absence of a high concentration of BIBR 1532 (50 μM).Figure 7 demonstrates that 50 μM BIBR 1532 did not affect MDA-MB 231 apoptosis except in cells grown in 0 g/l of glucose. Caspase-3 was not expressed in MCF-7 cells, and this result confirmed several reports that indicated caspase-3 deficiency in this cell line.

Caspase-3 expression was not detected in the Saos-2 cell line incubated at different glucose concentrations or treated with BIBR 1532. (Data not shown).

**Figure 8:**
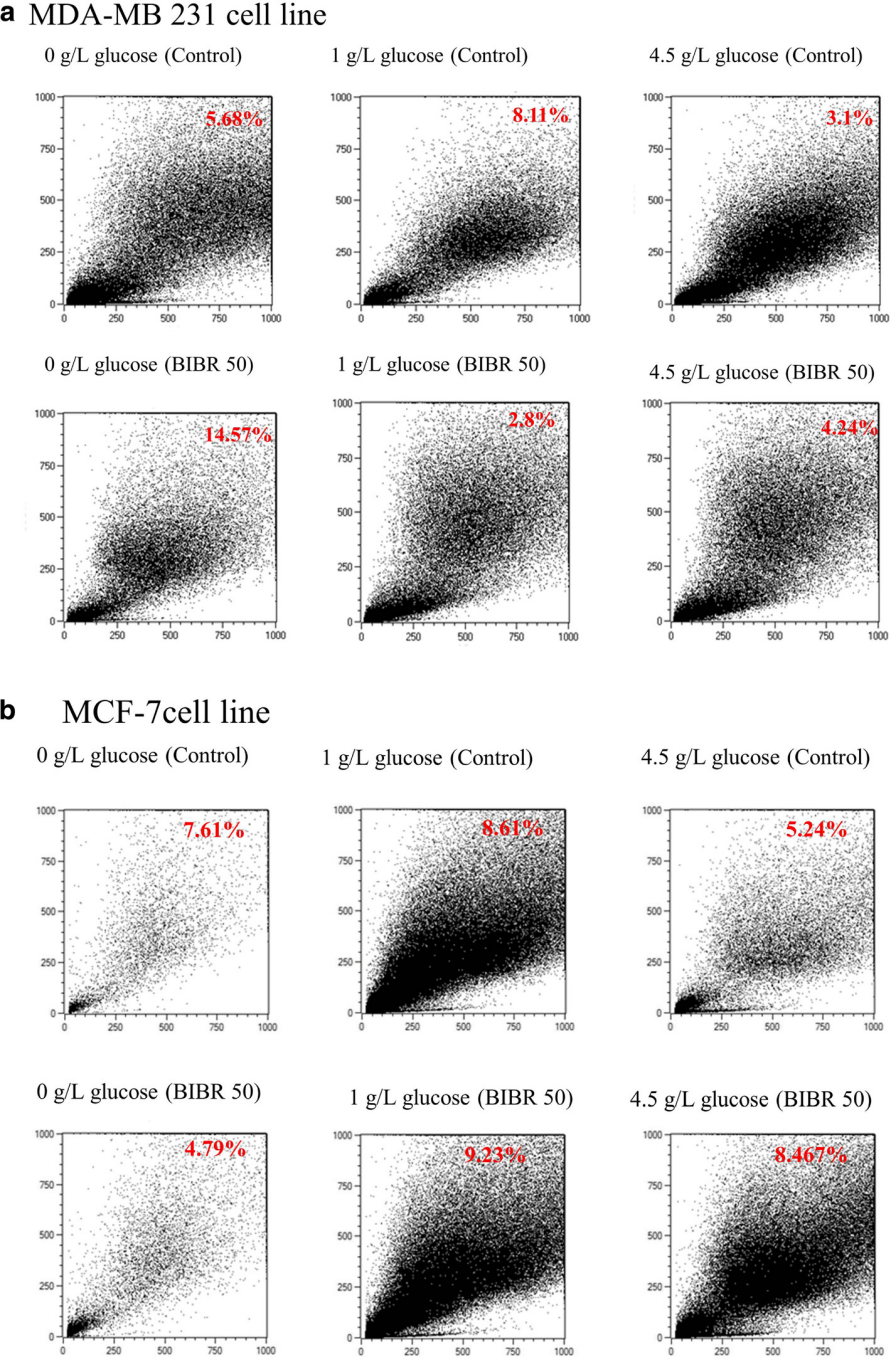
**FACS analysis of BIBR 1532-treated a. MDA-MB 231 and b. MCF-7 cells.** Cells were treated with 50 μM BIBR 1532 for 48 h and subsequently stained for Annexin-V and PI. The results represent three experiments.

The results obtained from this experiment suggest that caspase-3 is involved in the induction of apoptosis in BIBR 1532-treated MDA-MB 231 cells that are deprived of glucose. Apoptosis induction was also investigated via annexin V staining. Figure 8 and Tables 1 and 2 shows same results displayed in Figure 7. The percentage of annexin V-positive cells significantly increased in treated cells with BIBR 1532 without glucose compared to the control cells. These data suggest that the growth inhibitory effect of BIBR 1532 on MDA-MB 231 cells is partly due to its effect on apoptosis induction under restricted glucose conditions. In the MCF-7 cell line, high concentrations of BIBR 1532 did not elicit apoptosis as observed in MDA-MB 231, unless cells incubated in high glucose concentration (4.5 g/L) are treated with 50 μM BIBR 1532, as shown in Figure 8b and Tables 1 and 2.

**Table 1:** Percentage of apoptotic MDA-MB 231 cells treated with high BIBR 1532 concentration cells

**Cell line**	**Treatment**	**% of apoptotic cells**
MDA 0 g/L	Control	5.68%
MDA 0 g/L	BIBR 1532 (50 μM)	14.57%
MDA 1 g/L	Control	8.11%
MDA 1 g/L	BIBR 1532 (50 μM)	2.8%
MDA 4.5 g/L	Control	3.1%
MDA 4.5 g/L	BIBR 1532 (50 μM)	4.24%

(n = 3, mean ± SD).

**Table 2:** Percentage of apoptotic MCF-7 cells treated with high BIBR 1532 concentration

**Cell line**	**Treatment**	**% of apoptotic cells**
MCF-7 0 g/L	Control	7.61%
MCF-7 0 g/L	BIBR 1532 (50 μM)	4.79%
MCF-7 1 g/L	Control	8.61%
MCF-7 1 g/L	BIBR 1532 (50 μM)	9.23%
MCF-7 4.5 g/L	Control	5.24%
MCF-7 4.5 g/L	BIBR 1532 (50 μM)	8.46%

(n = 3, mean ± SD).

## Discussion

Due to its major role in cell immortalization, telomerase has become an attractive target for selective cancer therapy. Several approaches have been studied to decrease or completely inhibit the activity of telomerase. Among these efforts, we mention the direct and indirect inhibition of telomerase, immunotherapy using hTERT as a tumor-associated antigen, and gene therapy with a promoter-driven suicide promoter [27]. Because its expression is mainly restricted to tumor cells and it is the rate-limiting subunit in telomerase activity, hTERT has become the main target of inhibition to stop tumor growth. However, these efforts directly face a major obstacle described by the lag phase between the initiation of telomerase inhibition and the impact on the proliferation capacity [29,30]. Therefore, tumor cells with long telomeres will continue to grow upon telomerase inhibition until substantial telomere erosion has occurred. This limitation negatively impacts the application of this inhibition in patients with a high tumor mass. Out of several telomerase inhibitors, two molecules have been extensively studied: BIBR 1532 (2-((E)-3-naphtalen-2-yl-but-2-enolylamino)-benzoic acid) and Costunolide. The beneficial effect of caloric restriction, especially GR, on life span extension and cancer prevention and treatment has been very well documented. However, cell metabolic changes during this stress, especially on the enzymes activities, are not completely understood [3].

In the present study, we investigated the effects of BIBR 1532 on cell proliferation, mitochondrial metabolism, and apoptosis in MDA-MB 231 and MCF-7 cells under restricted glucose conditions. Furthermore, the role of GR on the telomerase activity and cell division rate was assessed for extended periods.

Our *in vitro* study showed that cell glucose deprivation dramatically decreases the telomerase mRNA expression and activity and cell proliferation. The important role of telomerase in cancer cell immortalization agrees with previous studies that reported a correlation between caloric restriction and cancer prevention and treatment. The cells incubated in 0 g/l of glucose were actually exposed to 0.65 g/l from FBS, which perfectly matches the physiological glycaemia in fasting state, whereas 1 g/l glucose mimics a postprandial state and 4.5 g/l mimics non-controlled glycaemia in diabetic patients or constant high glucose consumption. The increased telomerase activity in the presence of high glucose concentrations could at least partly explain the association between diabetes and cancer predisposition [31,32].

Despite the similar hTERT expression in cells grown in 1 and 0 g/l of glucose, a complementary experiment involving the cell count showed different results. In fact, cells grown in media containing 4.5 and 1 g/l of glucose were relatively similar, with minor significant differences in the cell number compared to cells grown in 0 g/l of glucose.

To confirm the role of telomerase on mitochondrial metabolism, a WST-1 assay was performed on a cell line negative for human telomerase (Saos-2). The same pattern in the mitochondrial metabolism that was observed for MDA-MB 231 and MCF-7 cells was found in the osteosarcoma cell line Saos-2. The same experiments were extended to investigate the effect of BIBR 1532 under restricted glucose conditions. We began by examining the effect of this inhibitor on cell proliferation. The MDA-MB 231 cell number reduced to almost zero when the cells were treated with BIBR 1532. These results could be explained by an increased vulnerability of telomerase to its inhibitor in response to GR.

Mitochondria are important organelles specified in energy production and cellular calcium homeostasis, and the capacity to release apoptotic proteins (members of the Bcl-2 family) is of important consideration [33]*.* Moreover, mitochondria are considered to be a major intracellular source of ROS. Succinate dehydrogenase (SDH), also known as quinone oxidoreductase, is a key regulator of metabolism and aerobic energy production. It couples the oxidation of succinate in the citric acid cycle to the reduction of ubiquinone in the electron transport chain, allowing cells to rely more on mitochondrial oxidative phosphorylation (OXPHOS) than aerobic glycolysis. Thus, this enzyme could reflect the activity of the mitochondrial respiratory chain [34]. Cell oxidative stress is produced with increased OXPHOS. These ROS have been implicated as second messengers in multiple signaling pathways, especially in apoptosis, as indicated by many reports [35]. We suggest that cells become more dependent on the Warburg effect than on OXPHOS after reducing the dehydrogenase activity via BIBR 1532 in 1 and 4.5 g/L of glucose. Aerobic glycolysis allows cells to rapidly multiply and contributes to ROS detoxification because carbons are diverted into the pentose phosphate pathway (PPP). This mechanism helps cancer cells survive under oxidative stress [36].

In MDA-MB 231, a high BIBR 1532 concentration did not stimulate apoptosis, as assessed by caspase-3 measurement, except for cells grown in 0 g/l of glucose independently of SDH activity modulation. This result could be explained by the extra-telomeric function of telomerase, i.e. its anti-apoptotic role [37]. The remaining telomerase activity (~20%) could sustain the proliferative capacity of cells grown in low glucose. The additive reduction activity of telomerase enhanced by BIBR 1532 treatment rendered cells more susceptible to apoptotic signals triggered by this inhibitor and was sufficient to induce caspase-3 expression in MDA-MB 231 cells. The absence of apoptosis mediated by the telomerase inhibition in MCF-7 cells is likely due to the up-regulation of hTERT by the estrogen receptor [38]. Further studies should be conducted to examine the effect of BIBR 1532-mediated telomerase inhibition in the MCF-7 cell line.

Our results again suggest the importance of GR to inhibit telomerase action; an additive effect between GR and telomerase inhibition by BIBR 1532 is also evident in apoptosis initiation in the triple-negative cell line MDA-MB 231.

## Conclusions

This report demonstrated that the telomerase inhibitor BIBR 1532 exhibits an apoptotic effect via telomerase inhibition that is potentiated by restricted glucose conditions.

## Materials and methods

### Cell culture and glucose restriction

The established human triple-negative breast carcinoma cells (MDA-MB 231), human-estrogen positive breast carcinoma cells (MCF-7) and human negative telomerase osteosarcoma cells (saos-2) were purchased from ATCC (American Type Culture Collection). All MDA-MB 231, MCF-7, and Saos-2 cells were cultured in DMEM (Dulbecco’s modified eagle’s media, Sigma-Aldrich) at different glucose concentrations (0, 1 and 4.5 g/l) and supplemented with 10% FBS (fetal bovine serum, Sigma-Aldrich, Taufkirchen, Germany), 1% Pen-Strep (penicillin-streptomycin, Sigma-Aldrich, Taufkirchen, Germany) and 2 mM glutamine (Sigma-Aldrich, Taufkirchen, Germany) and maintained in a humidified atmosphere of 5% CO2 at 37°C. The cells were pre-incubated for five days in the appropriate media, and treatment occurred when cells reached 80% confluence.

### Cell count

MDA-MB 231 AND MCF-7 cells were seeded at very low densities in 25 cm^2^ flasks and grown in DMEM culture media containing 10% FBS and 1% Pen/Strep at different glucose concentrations with or without 2.5 μM of BIBR 1532. The medium was replaced every two days, and fresh telomerase inhibitor was added each time. At the end of each week, the cells were split and counted at least three times using bromophenol blue and a hemocytometer. Figures 3a and b show the effect of GR and treatment with 2.5 μM BIBR 1532 on MDA-MB 231 and MCF-7 cells by calculating the obtained cell number after each count on those seeded.

### Cell viability and mitochondrial metabolism assay

The cell proliferation was evaluated using WST-1 (Takara Bio Inc, Ostu, Shiga, Japan), a tetrazoluim salt, cleaved into formazan by succinate-tetrazolium reductase, which exists only in the mitochondrial respiratory chain and is active only in viable cells. The production of formazan is directly related to the cell number due to the active mitochondrial metabolism and to its absorbance, which was measured using an ELISA reader at 450 nm. In our study, cells grown in different glucose concentrations were seeded in 96-well plates at a density of 10^4^ cells per well and allowed to adhere for 24 hours, and the assay was performed following the manufacturer’s instructions.

### Telomerase activity assay

MDA-MB 231 and MCF-7 extracts, from 1.5 million cells for each condition, were prepared according to the manufacturer’s protocol using a Quantitative Telomerase Detection Kit (Allied Biotech, Inc Vallego, California, USA). Briefly, the cell pellets were washed twice with cold PBS, and the cells were then lysed with an appropriate volume of the provided lysis buffer. After 30 minutes of incubation on ice, the suspension was centrifuged for 30 min at 4°C and 12000 × g. The supernatant was then aliquoted to further assess the telomerase activity and protein concentration.

The telomerase activity was measured by Real-Time Polymerase Chain Reaction (PCR) (Applied Biosystem, USA), which is based on the ability of telomerase in the cell extracts to synthesize telomeric repeats onto an oligonucleotide substrate, and the resultant extended products are subsequently amplified by PCR. The generated PCR products were then visualized using the highly sensitive DNA fluorochrome SYBR Green. The PCR products were detected following the binding of SYBR Green dye to double-strand DNA.

### Detection of hTERT and hTR gene expression

Total RNA from MDA-MB 231 and MCF-7 cells was extracted using THE QIAamp RNA extraction Kit (Qiagen Inc., Valencia, CA, USA). Complementary DNA (cDNA) was synthesized from 0.1 to 2.5 μg of total RNA in a 20-μl reaction using the Omniscript Reverse Transcription Kit (Qiagen Inc., Valencia, CA, USA). The expression of hTERT and GAPDH was assessed using the multiplex hTERT/GADPH PCR technique and specific primers. The primers were defined as follows: hTERT: 5′-CGGAAGAGTGTCTGGAGCAA-3′, 5′-GGATGAAGCGGAGTCTGGA-3′; hTR: 5′-GCGGAAGACAGTGGTGAACT-3′, 5′-AGCTGGAGTAGTCGCTCTGC-3′; GAPDH (Glyceraldehyde 3-phosphate dehydrogenase): 5′-TGGGATGGACTGTGGTCATGAG-3′, 5′-ACTGGCGTCTTCACCACCATGG3-′, which was used as the internal control. PCR amplification was performed using Dream Taq Green PCR 2× Master Mix (Fermentas, USA), and the reactions were subjected to 40 PCR cycles (BioRad, Germany) of 94˚C for 30 seconds, 60˚C for 30 seconds, and 70˚C for 30 seconds followed by 7 minutes of extension at 72˚C. The PCR products were separated via 1.2% agarose gel electrophoresis and visualized by ethidium bromide staining using the UVP BioDoc system (UVP, England).

### Protein quantification

The protein concentration was measured with the Bradford method according to the manufacturer’s recommendations using an acidic solution of Coomassie Blue G-250 dye, which is a reagent in the Bio-Rad Protein Assay, and BSA, which served as the standard. Serial dilutions of BSA at known concentrations were prepared. Five milliliters of Coomassie Blue solution (5× diluted) were added to the diluted proteins. The absorbance of the colored solution was measured with a spectrophotometer at 595 nm.

### Caspase-3 apoptosis assay

The caspase-3 level was determined using a caspase-3 (active) human ELISA Kit from Invitrogen. Briefly, MDA-MB 23, MCF-7 and saos-2 cells were seeded in 6-well plates in DMEM at different levels of GR and treated with 50 μM BIBR 1532. After 48 hours of treatment, the cells were collected in cold phosphate buffered saline 1 × (PBS) and lysed with cell extraction buffer (10 mM Tris, pH 7.4, 100 mM NaCl, 1 mM EDTA, 1 mM EGTA, 1 mM NaF, 20 mM Na_4_P_2_O_7_, 2 mM Na_3_VO_4_, 1% Triton X-100, 10% glycerol, 0.1% SDS, 0.5% deoxycholate, 1 mM PMSF and protease inhibitor cocktail). The cell extracts were then used to determine the concentration of caspase-3. The ELISA method was based on the fixation of cleaved caspase-3 at Asp 175/Ser 176 between two antibodies. This enzyme was detected after a detection antibody bound to the second antibody, followed by adding a substrate solution that was acted upon by the bound enzyme to produce color. The O.D. (optical density) of this colored product was measured at 450 nm with an ELISA Reader (Thermo Scientific, USA), and this O.D. is directly proportional to the concentration of human active caspase-3 present in the sample.

### Annexin V-FITC apoptosis assay

The FITC annexin V apoptosis detection kit (*Abcam*) was used following the manufacturer’s instructions. MDA-MB 231 and MCF-7 cells grown in different glucose concentrations were plated at a density of 2 × 10^5^ cells/well on six-well plates. After 48 h of treatment with 50 μM BIBR 1532, the cells were trypsinized and collected in ice cold PBS. The cells were washed twice with cold PBS and re-suspended in 500 μL binding buffer containing 5 μL Annexin V-FITC. The signals were detected using a FACScan flow cytometer (MACSquant, Germany) with excitation and emission settings of 452 nm.

### TERT siRNA transfection

MDA-MB 231 and MCF-7 cells were maintained in DMEM at different concentrations of glucose and plated in 96-well plates at a density of 2 × 10^4^ cells per well. Prior to seeding, 5 μL of specific siRNA, Hi-perfect (*Qiagen*) and serum-free media were added into each well and incubated for 10 minutes at ambient temperature. One hundred microliters of cell suspension were added to each well to initiate a treatment of 72 h. Two siRNAs were used in this assay; the first was TERT3, which targets the mRNA of the sub-catalytic unit of telomerase and the second was Death, which targets expressed human genes that are indispensable for cell survival. This siRNA was used as a positive control because it induces cell death that was visible by light Microscopy. The siRNA sequences were designed with the RNAi Human/Mouse Starter Kit (Qiagen Inc., Valencia, CA, USA). For hTERT, the siRNA sense sequence was 5′-GGAGCAAGUUGCAAAGCAUTT-3′ and the anti-sense was 5′-AUGCUUUGCAACUUGCUCCAG-3′, the target sequence was 5′-CTGGAGCAAGTTGCAAAGCAT-3′. Transfection monitoring was assured by the cell death control siRNA, which is a blend of siRNAs that target human genes essential for cell survival.

### Statistical analysis

The statistical analysis was performed with *t*-test using the online statistical software Graph-Pad Quickcalcs (http://www.graphpad.com/quickcalcs/ttest1.cfm).

### Ethical committee

This project was approved by the Ethical Committee of the Faculty of Medicine of Saint-Joseph University, number 53/2012.

## Abbreviations

CR: Caloric restriction; GR: Glucose restriction; hTERT: Human telomerase reverse transcriptase; hTR: Human template RNA; ROS: Reactive oxygen species; SDH: Succinate dehydrogenase; ER: Estrogen receptor; PR: Progesterone receptor.

## Competing interests

We state here that none of our authors has financial or other competing interests that might be construed as influencing the results or interpretation of our study.

## Authors’ contributions

LW carried out the cell culture, the telomerase activity measurement, and the mitochondrial metabolism experiments and drafted the paper. NA, IR and RS contributed to the design of the study, drafted the paper and contributed to the discussion. RS performed the ELISAs experiments. CK performed the flow cytometry experiments. GH proposed the original idea of this study, designed the experiments and drafted the paper. All authors read and approved the final manuscript.
